# A cross-sectional bibliometric analysis of care phases in SCImago’s top five intensive care journals in 2012 and 2022

**DOI:** 10.3389/fmed.2025.1633371

**Published:** 2025-09-09

**Authors:** Mark Z. Y. Tan, Pei Shan Lim, Saarth Shiralkar, Bruce Liu, Laura Scott, Matthew Palethorpe, Paul Dark, Jan Hansel

**Affiliations:** ^1^The University of Manchester, Manchester, United Kingdom; ^2^North West School of Anaesthesia, Manchester, United Kingdom; ^3^North West School of Intensive Care Medicine, Manchester, United Kingdom; ^4^Northern Care Alliance NHS Foundation Trust, Salford Care Organisation, Manchester, United Kingdom

**Keywords:** critical illness, intensive care, emergency care, bibliometric analysis, NIHR James Lind Alliance, post-intensive care syndrome, SCImago

## Abstract

**Background:**

The critically ill patient’s journey can broadly be divided into three phases; events prior to admission, during the ICU stay, and following discharge. There is a paucity of evidence on what proportion of research outputs in high-impact intensive care journals address the respective phases. We aimed to investigate how intensive care research published in SCImago’s top five journals mapped onto phases of care and the National Institute of Health and Care Research James Lind Alliance (NIHR JLA) Priority Setting Partnership research topics over a decade. The study was prospectively registered with the Open Science Framework https://osf.io/r8vs5/.

**Methods:**

We conducted a bibliometric cross-sectional analysis of intensive care research published in 2012 and 2022, in the top five intensive care journals according to the SCImago ranking system. We categorised studies according to phases of care: “pre-ICU”, “in ICU”, “post-ICU”, or “multiple phases”; and according to the previously published NIHR JLA research topics. We adhered to relevant PRISMA and BIBLIO reporting guidelines in conducting the review.

**Results:**

In 2012 and 2022, respectively, 755 (73%) and 602 (73.7%) studies were focused on the “in ICU” phase, 148 (14.3%) and 87 (10.6%) studies on the “pre-ICU” phase, and 39 (3.8%) and 56 (6.9%) studies on the “post-ICU” phase. In both years, two NIHR JLA research topics were most represented: “respiratory/ventilation” (19.1% and 26.6% in 2012 and 2022, respectively) and “infection/sepsis” (15.6% and 13.7% in 2012 and 2022, respectively).

**Conclusion:**

Among the top five intensive care journals according to the SCImago ranking system, research tends to be centred on the period during patients’ ICU stay, and characterised most by the topics of “respiratory/ventilation” and “infection/sepsis.”

## 1 Introduction

Critical illness patient journeys are marked by non-linear trajectories which begin before admission to the ICU and continue far beyond discharge from hospital. Before admission to the ICU, social determinants and existing health inequalities predispose patients to critical illness and poorer outcomes (1, 2). Upon discharge, the multi-faceted post-intensive care syndrome (PICS), experienced by up to 80% of ICU survivors, demonstrates lasting effects of critical illness on the affected individual, their family, and society far beyond the hospital stay (3, 4). The critical illness patient journey can thus be divided into three distinct phases: “pre-ICU”, “in ICU”, and “post-ICU”. There is a paucity of published evidence examining the representation of research according to these phases.

Bibliometric analysis is an increasingly employed, robust method of gaining insights into the landscape of published literature. It has been used to explore the range of a particular topic, map the connections between research groups, and identify emerging trends (5, 6). It has also been used to support research funding decisions and in priority-setting exercises (7). Within intensive care, for example, bibliometric analysis has been used to identify the most influential papers (8), understand the scope of a particular condition (9), or track an emerging trend such as artificial intelligence (10). Bibliometric analysis has not yet been used to investigate the research landscape across phases of care in critical illness. One of the topics that has been analysed using bibliometric methods is PICS (9), through identifying key research groups and locations, presenting keyword co-occurrences (an indication of key topics), and citation metrics.

Notably, PICS is just one out of eighteen research topics identified as a key priority by the UK’s National Institute for Health and care Research (NIHR) James Lind Alliance (JLA) priority setting partnership (11). Using a range of consensus techniques and involving a variety of stakeholders (including patients, clinicians, and researchers), the NIHR JLA process both categorised the range of intensive care research into topics and highlighted the top ten priorities for future research. Two of the top three priorities specifically correspond with pre-ICU and post-ICU phases of care (“early identification and escalation of care”, and “supporting recovery from critical illness”, respectively). The NIHR JLA process has resulted in increased empowerment and involvement in research at patient, practitioner, and policymaker levels (12), however, its impact on research has not yet been fully explored. Thus, the NIHR JLA topics provide a useful framework in conjunction with the phases of care, with which to analyse high-impact intensive care research.

The primary objective of this study is to characterise the distribution and evolution of intensive care research over a decade by mapping publications in leading journals to phases of the patient journey and NIHR JLA research priorities, and by analysing trends in quantity, performance/impact, and structure. The secondary objective is to identify research gaps in JLA topics in critical care.

## 2 Materials and methods

This was a collaborative study performed by the Northwest Research and Audit Group (NWRAG). NWRAG is a trainee-led research group spanning the specialties of anaesthesia and intensive care medicine, operating across the Northwest of England. We followed PRISMA and BIBLIO guidelines for bibliometric analyses (13, 14) (Supplementary Appendix 1, 2). The study was prospectively registered with the Open Science Framework^1^. There were no deviations from the original protocol.

We conducted a cross-sectional search and bibliometric analysis of all original research published in 2012 and 2022 in any of the top five ICM journals according to the SCImago Journal Ranking system. This system uses Scopus, the largest public scientific database, and ranks journals based on a variety of metrics including journal coverage, citation metrics and networks (15).

We searched PubMed (NIH), Scopus (Elsevier), and Web of Science (Clarivate) databases in February 2023. The search strings are presented in Supplementary Appendix 3. We included original research focused on intensive care (Supplementary Appendix 4). Perspectives, opinions, letters, comments, or editorials were excluded, as were manuscripts not related to critical illness. Educational research was excluded. We did not exclude records based on language, however, as all included journals are published in English. This was a non-interventional analysis, therefore, we did not undertake a formal risk of bias assessment. At study commencement, 2022 was the first full chronological year available for analysis. To analyse progress and the impact of the JLA exercise on the spread of published research topics, we also analysed papers from 2012, providing a 10-year gap between the two cross-sections and a period prior to the publication of the JLA priorities. As the study aimed to demonstrate the spread of research, we opted to include paediatric and neonatal intensive care research too, since they are recognised as under-represented and under-researched populations (16).

**FIGURE 1:**
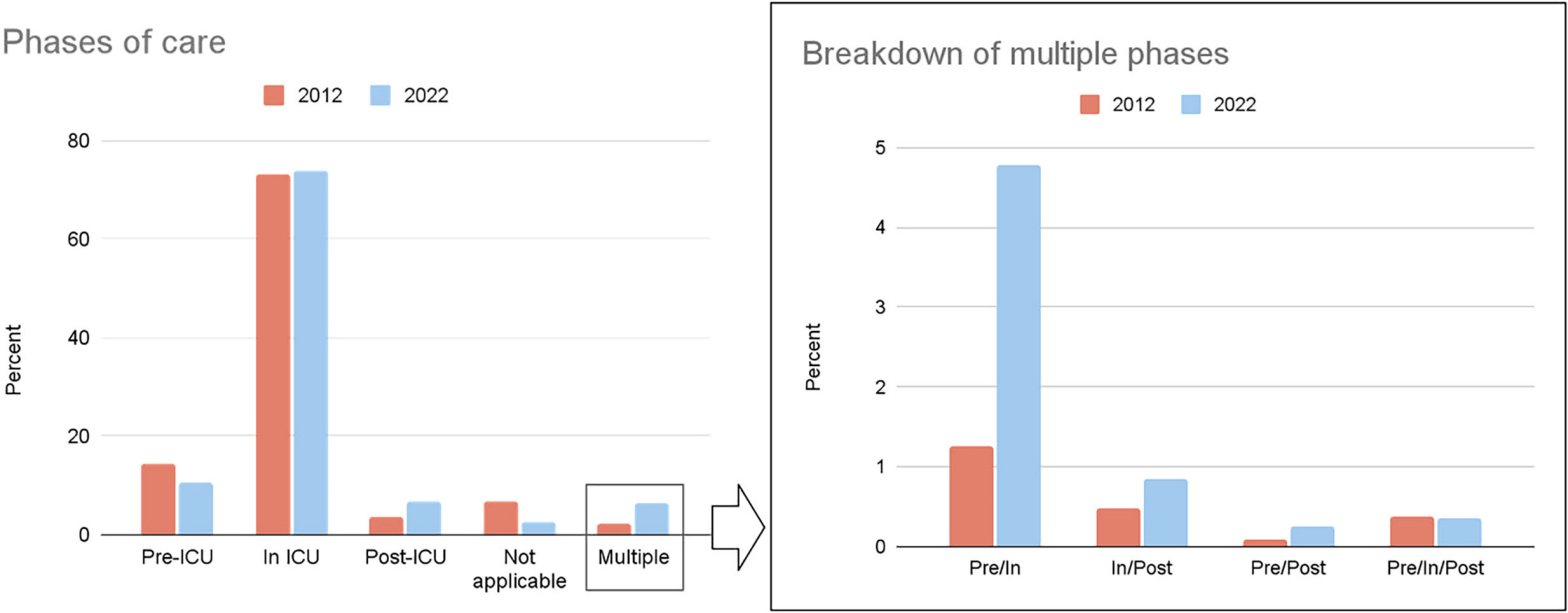
PRISMA diagram from 2012 and 2022.

We used the Rayyan screening software (17) for a three-stage screening process (18), which was carried out by MT, SS and JH. Most records underwent single assessor screening due to the volume of data, but cross-checking occurred for a random sample to ensure dependability (19). Uncertainties were resolved through double-blinded screening by a second reviewer and any further disagreements were resolved through screening by a third reviewer. The PRISMA flow diagram is shown in Figure 1.

**FIGURE 2:**
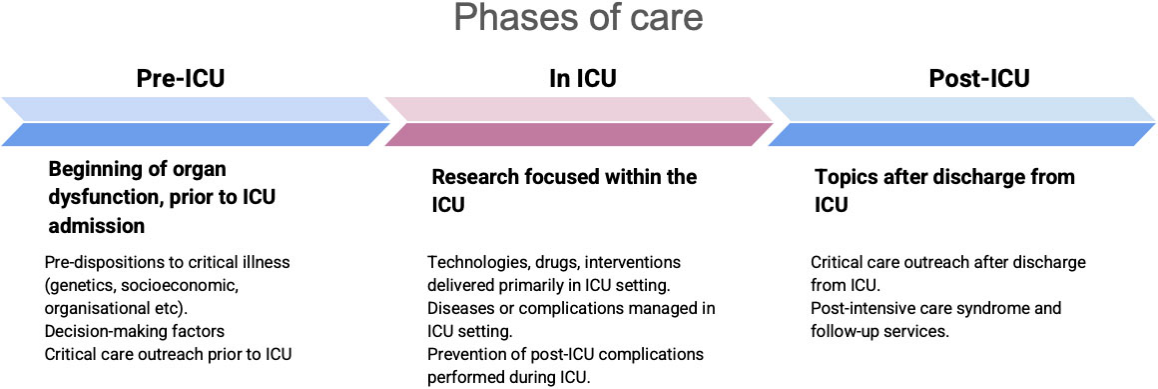
Boundaries of phases of care.

A data extraction table was developed *a priori* and further refined with a pilot selection of ten random papers which met inclusion and exclusion criteria. The full dataset is openly published and accessible at https://zenodo.org/records/10696000. We assessed all studies for their predominant focus in the patient’s critical illness journey. We coded studies according to the phases of care as follows: prior to ICU admission (“pre-ICU”), during the ICU admission (“in ICU”), and following ICU admission (“post-ICU”). All authors undertook data extraction. One author (MT, PS, SS, MP, LS, JH) coded each record, according to the boundaries listed in Figure 2; due to the volume of data, double coding was felt to be impractical. To mitigate this, an independent blinded senior author cross-checked a random sample of included studies (19). Several studies included multiple phases; this was handled by coding according to the permutations “pre/in”, “pre/post”, “in/post”, and “pre/in/post-ICU”. To categorise studies according to research topic, we made use of the NIHR JLA list of topics from their priority setting partnership for intensive care research (11). All uncertainties in phases or research topics were settled through discussion with an additional independent reviewer. Citations were analysed according to phase of care, with medians and interquartile ranges (IQR) reported, and statistically compared using the Kruskal-Wallis rank-sum test. Comparisons were plotted between citations and Altmetric scores for all included studies, with statistical comparisons made using the Pearson correlation coefficient (r). All statistical analyses and plots were generated in R (version 4.3.1).

## 3 Results

According to the SCImago ranking system, the top five intensive care journals in 2022 were (1) American Journal of Respiratory and Critical Care Medicine (American Thoracic Society), (2) Intensive Care Medicine (Springer Verlag), (3) Critical Care (BioMed Central Ltd.), (4) Critical Care Medicine (Lippincott Williams and Wilkins Ltd.) and (5) Annals of Intensive Care (Springer Verlag). In 2012, these were (1) American Journal of Respiratory and Critical Care Medicine, (2) Critical Care Medicine, (3) Intensive Care Medicine, (4) Critical Care, and (5) Annals of Intensive Care.

**FIGURE 3:**
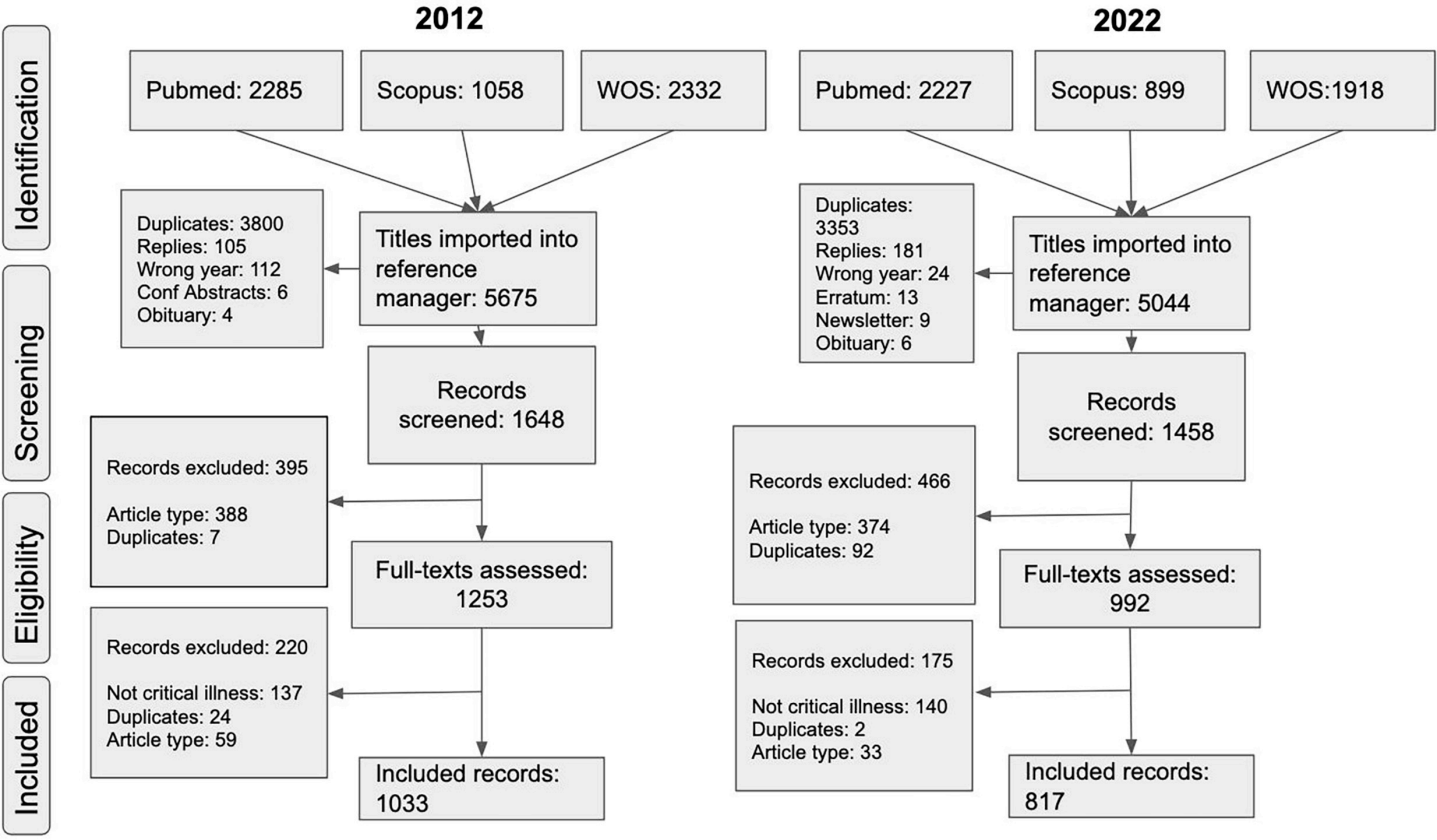
Studies according to phases of care.

**FIGURE 4:**
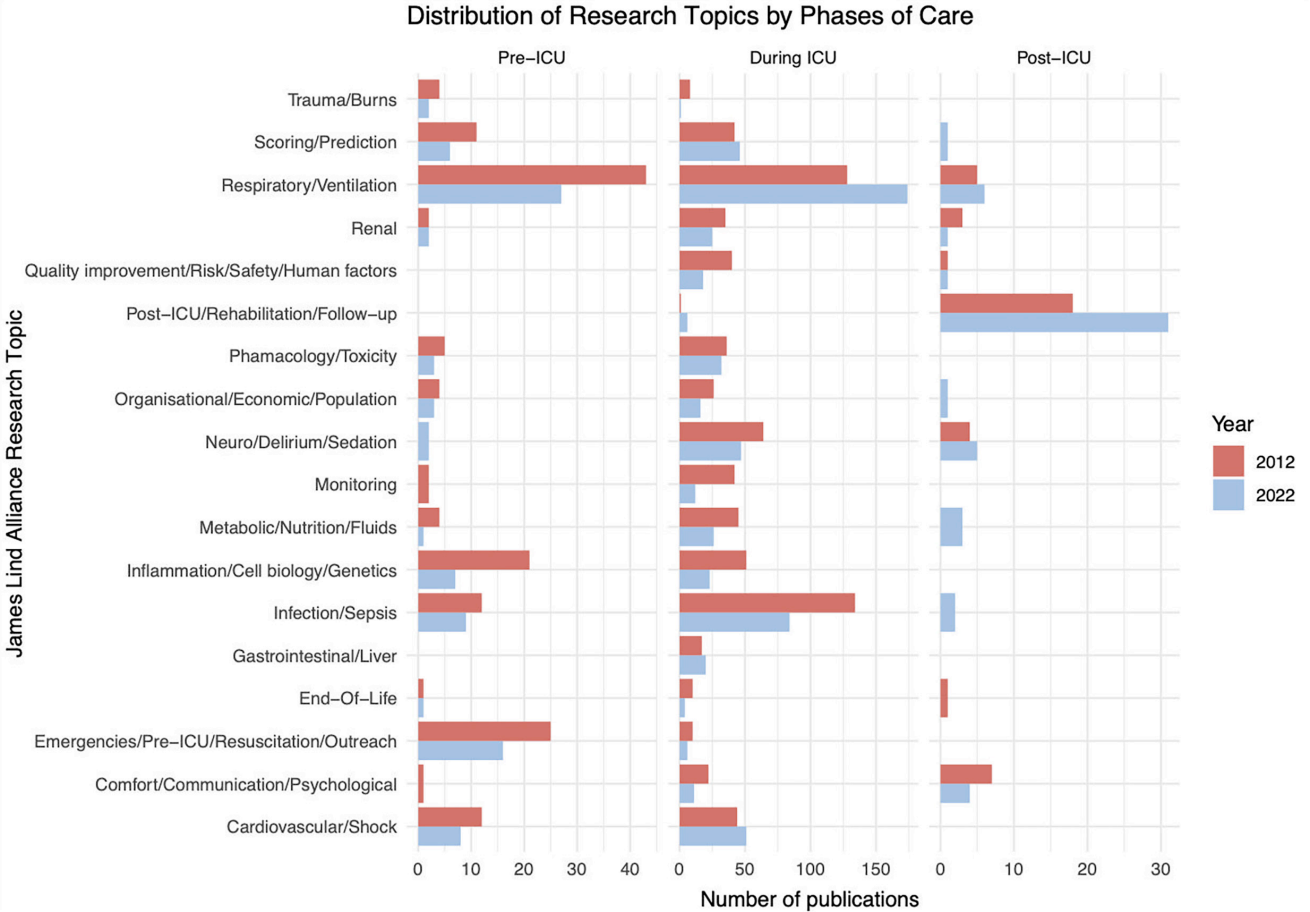
Studies categorised according to the NIHR JLA research topics and phases of care.

We identified 5675 and 5044 records from 2012 and 2022, respectively. After deduplication, 1033 and 817 records were included from each year. Table 1 shows the characteristics of included studies, the number and proportion of different study types, populations, phases of care (Figure 3), and research topic according to the NIHR JLA framework (Figure 4 and Supplementary Appendix 5).

**TABLE 1:** Characteristics of studies and findings.

	2012, *n* (%)	2022, *n* (%)
**Research type**		
Review	125 (12.1)	80 (9.8)
Systematic review & meta-analyses	53 (5.1)	65 (8.0)
Intervention	140 (13.5)	139 (17.0)
Observation	538 (52.0)	505 (61.8)
Non-human	178 (17.2)	28 (3.4)
**Study population**		
Adult	609 (58.9)	667 (81.6)
Paeds	83 (8.0)	35 (4.3)
Neonates	9 (0.9)	4 (0.5)
Not specified	139 (13.4)	73 (8.9)
Animals	165 (16.0)	22 (2.7)
Multiple	29 (2.8)	16 (2.0)
**Time domain**		
Pre-ICU	148 (14.3)	87 (10.6)
In ICU	755 (73.0)	602 (73.7)
Post-ICU	39 (3.8)	55 (6.7)
Not applicable	69 (6.7)	21 (2.8)
Multiple: pre/in ICU	13 (1.3)	38 (4.7)
Multiple: in/post-ICU	5 (0.5)	8 (1.0)
Multiple: pre/post-ICU	1 (0.1)	2 (0.2)
Multiple: pre/in/post-ICU	4 (0.4)	4 (0.5)
**NIHR James Lind Alliance research topic**		
Emergencies/pre-ICU/resuscitation/outreach	38 (3.7)	27 (3.3)
Infection/sepsis	161 (15.6)	112 (13.7)
Gastrointestinal/liver	20 (1.9)	21 (2.6)
Inflammation/cell biology/genetics	86 (8.3)	41 (5.0)
End-of-life	13 (1.3)	6 (0.7)
Cardiovascular/shock	62 (6.0)	60 (7.3)
Monitoring	44 4.3)	14 (1.7)
Renal	44 (4.3)	30 (3.7)
Pharmacology/toxicity	49 (4.7)	37 (4.5)
Respiratory/ventilation	197 (19.1)	217 (26.6)
Scoring/prediction	54 (5.2)	57 (7.0)
Trauma/burns	12 (1.2)	4 (0.5)
Metabolic/nutrition/fluids	49 (4.7)	32 (3.9)
Comfort/communication/psychological	30 (2.9)	16 (2.0)
Neuro/delirium/sedation	70 (6.8)	58 (7.1)
Organisational/economic/population	41 (4.0)	25 (3.1)
Post-ICU/rehabilitation/follow-up	19 (1.8)	37 (4.5)
Quality improvement/risk/safety/human factors	45 (4.4)	23 (2.8)
**Total**	**1034 (100)**	**817 (100)**

We noted a decrease in the proportion of non-human studies from 2012 to 2022, from 17.2 to 3.4%, corresponding with a similar decrease in animal studies from 16% in 2012 to 2.7% in 2022. There was also an increase in adults as the main study population from 58.9% in 2012 to 81.6% in 2022. Consequently, the proportion of paediatric research almost halved between 2012 to 2022, from 8 to 4.3%. While the focus on the phase “in ICU” remained the same between the years, there has been an increase in proportion of the “post-ICU” phase from 3.8% in 2012 to 6.9% in 2022.

Considering the NIHR James Lind Alliance research topics, there was a broad spread, however, two topics were outstanding from both years: “respiratory/ventilation” and “infection/sepsis” (Figure 4, Table 1 and Supplementary Appendix 5). There was an increase in studies on “respiratory/ventilation” from 197 (19.1%) in 2012 to 217 (26.6%) in 2022. We identified 161 (15.6%) studies on “infection/sepsis” in 2012 and 112 (13.7%) in 2022. These topics were most represented in the “pre-ICU” and “in ICU” phases (Figure 4). All other topics represented less than 10% of all research, including twelve out of the eighteen topics which were represented by less than 5% of all published research each. The “emergencies/pre-ICU/resuscitation” topic encompassed 38 (3.7%) studies in 2012 and 27 (3.3%) studies in 2022. Notably, despite a considerable increase in a decade, “post-ICU/rehabilitation/follow-up” research topics only consisted of 19 (1.8%) studies in 2012 and 37 (4.5%) in 2022.

The median (IQR) number of citations were 52.3 (6–67), 30.6 (5–33.3) and 40.8 (3.8–39) for the “pre-ICU”, “in ICU” and “post-ICU” phases, respectively. The scatterplot of number of citations according to phase of care is presented in Supplementary Appendix 6. We noted a statistically significant difference between the three phases with X^2^ value of 17.24 (*P* < 0.001). There was poor correlation between citations and published Altmetric scores (Pearson *r* = −0.0218, *P* = 0.3993) with an apparent inverse correlation in the extremes with the most well-cited studies attaining low Altmetric scores and vice versa (Supplementary Appendix 7).

## 4 Discussion

Several findings are notable from this bibliometric analysis of intensive care research from 2012 and 2022. From the included journals, most research (73%) is centred in the ICU phase of care. Only 14.3% and 10.6% of studies were focused on the phase of “pre-ICU” in 2012 and 2022, respectively (also corresponding to the low proportion of studies in “emergencies/resuscitation” and “organisational/economic/population” health topics from 2012 to 2022). The proportion of studies looking at the “post-ICU” phase increased from 3.8% in 2012 to 6.7% in 2022 (corresponding to the increase in “post-ICU/rehabilitation/follow-up” topic) (Figure 4). Despite these findings, two research topics continue to dominate: “infection/sepsis” (15.6% in 2012 and 13.7% in 2022) and “respiratory/ventilation” (19.1% in 2012 and 26.6% in 2022) (Figure 4 and Table 1). The change in funding priorities during the COVID19 pandemic may account for some of the increase in respiratory research (20).

### 4.1 Critical illness phases in context

There has been an increase in studies covering multiple phases, from 2.3% in 2012 to 6.3% in 2022. One study in Spain analysed how multi-pronged improvements in care have resulted in reduced mortality in septic patients with multi-organ failure (21). It highlighted how advancements in the “pre-ICU” and “in ICU” phases have resulted in improved long-term outcomes in the “post-ICU” phase. Another study encompassing the “pre-ICU”, “in ICU”, and “post-ICU” phases focused on the impact of premorbid mental health issues on the ICU incidence of delirium and its impact on long-term quality of life (22). While it is not feasible for all research to be similarly structured, such studies may serve as valuable examples of how multiple phases of care can be investigated.

It is important to interpret these results in context. Some large, highly-cited, landmark studies in the field of intensive care research are published in general medical journals, such as New England Journal of Medicine (23), Lancet group of journals (24), and the Journal of the American Medical Association (25, 26). In 2022, these included studies such as REMAP-CAP (26), SUDDICU (25), and RECOVERY (24). Indeed, a previous bibliometric analysis of the most cited intensive care research revealed that papers were published across a range of journals, including those assessed in our study, as well as the aforementioned general medical journals (8). It is worth noting that the example landmark trials above all focus on the “in ICU” phase of care, and on the two dominant NIHR JLA topics. Other specialty journals, including nursing and allied health, also publish impactful output from the field of intensive care; in these journals, the representation of phases of care may differ. There are suggestions that research topics such as quality of life and long-term care may feature more frequently in such journals (27, 28). However, these previously performed bibliometric analyses were not designed to investigate the phases of care. Instead, they either focused on a particular topic of interest (e.g., PICS) (9), or a broad overview of an entire discipline (e.g., nursing).

Non-inclusion of general medical journals represents a considerable limitation to the broader generalisability of our work. The decision to focus on a subset of intensive care journals (in our case SCImago’s top five) represented a trade-off by balancing resources required for manual coding of each identified record. Currently available bibliometric software is not yet equipped with the tools to assign automated labels. However, large language model integration with limited supervision could represent an avenue for future work along a similar framework at a larger scale, thus offering broader insights into publication patterns according to bespoke discretionary criteria (e.g., phases of care). Presently, a drawback to our study remains the limited breadth of performance/impact indicators, such as h-indices or citations per year. Furthermore, we were limited in terms of science mapping, as the minimum dataset did not allow for bibliographic coupling.

### 4.2 Journal and manuscript ranking

The SCImago ranking system is one of several available journal-level metric systems (29). It ranks journals according to a citation-based calculation which accounts for time and status of the journal. Based on Scopus data, it captures most scholarly publications. Since it categorises journals based on subject, it is an indicator of the journals that produce some of the most impactful studies in each field. However, as it is constrained to specialties, it is unable to capture the full spectrum of intensive care research. In addition, there remain criticisms of the SCImago system, explored in depth elsewhere (29), which make it non-superior to other scientific ranking systems (citation count, Altmetrics, impact factor, or Eigenfactor). Therefore, an ideal scoring system does not yet exist, and this is a limitation of performing any bibliometric analysis.

Looking at article-level metrics, most studies that generated the highest Altmetric scores tended to have low citation scores, whereas those with the highest citation scores tended to have low Altmetric scores (Supplementary Appendix 7). The Altmetric score indicates how much attention a paper has received (30). It gathers data from a wide range of social media websites, video-streaming platforms, and news outlets. Three main foci (volume of mentions, differential weighting of sources, and author-centred metrics) are then used to calculate a score. The poor correlation between Altmetric and citation scores does not reflect a previously performed study in 2023, which highlighted that certain Altmetrics correlate well with citation counts, and both in turn also reflect the UK Research Excellence Framework 2021 quality indicators (31). They, however, also acknowledged that there are ongoing uncertainties regarding the interpretation of Altmetrics, that citation scores do not necessarily reflect scholarly impact, and that another study demonstrated poor correlations between certain Altmetrics and quality scores (32). Conversely, a recently published analysis of top nursing journal articles exploring the relationship between Altmetric attention scores and citations demonstrated a similar distribution to our study, with overall weak correlation coefficients (33). Thus, the observation of the opposing extremes of Altmetric scores and citation counts may warrant further study.

### 4.3 Limitations and strengths

There are some further methodological constraints to acknowledge in our work. Critical illness journeys themselves are non-linear; they are not neatly categorised into phases of care. The “pre-ICU”, “in ICU”, and “post-ICU” phases were a best-fit agreed amongst the authors and applied well to most studies. Similarly, some research spanned more than one NIHR JLA priority topic. For example, a study on healthcare spending on respiratory diseases fits into both the “organisational/economic/population” and the “respiratory/ventilation” topics (34). Similarly, a study on biomarkers to predict renal recovery could be categorised as “scoring/prediction” or “renal” (35). Again, a best-fit approach was adopted, but may not fully represent the scope of captured research.

Despite some of the limitations explored above, this study has several strengths. It affords a more granular and in-depth analysis of intensive care research compared to other bibliometric studies in this specialty (8–10), providing a richer contextual understanding. Such a preliminary overview is particularly important to help us better understand the ongoing needs and research gaps, alignment with wider agendas, and future research funding (36–38). Further studies, especially in the broader literature, may also provide a more comprehensive understanding of the landscape of intensive care research. In this regard, the framework presented in our study can be used as a tool for training large language models, which may in turn overcome some of the current limitations of more rigidly defined automated bibliometric analyses.

## 5 Conclusion

Among the top five intensive care journals according to the SCImago ranking system, research tends to be centred on the period during the patient’s stay in the ICU and dominated by the topics of “respiratory/ventilation” and “infection/sepsis” according to the NIHR JLA framework. Whilst there have been some improvements in the representation of the post-ICU phase in this subset of journals, further work is required to understand the representation of phases of care in other medical, nursing, and allied health journals. The taxonomy and framework used in this study may be used both for future bibliometric analyses, and as a tool to train large language models for this purpose. By highlighting the current landscape, researchers, clinicians, patients and other relevant stakeholders can better understand research strengths, needs and trajectories.

## Data Availability

The datasets presented in this study can be found in online repositories. The names of the repository/repositories and accession number(s) can be found below: https://zenodo.org/records/10696000.

## Footnotes

1
https://osf.io/r8vs5/
