# Supplementary material for: A cross-sectional bibliometric analysis of care phases in SCImago’s top five intensive care journals in 2012 and 2022
Source: Front Med (Lausanne). 2025 Sep 9;12:1633371. doi: 10.3389/fmed.2025.1633371 (PMC12454406; doi:10.3389/fmed.2025.1633371)
Supplement: Supplementary file 1 [file Data_Sheet_1.docx]

Supplementary Material

Appendix S1 – PRISMA Checklist

Appendix S2 – BIBLIO Checklist

Appendix S3 – Search strings

Appendix S4 – Inclusion and exclusion criteria

Appendix S5 – Studies categorised according to James Lind Alliance research topics

Appendix S6 – Citations by phase of care

Appendix S7 – Scatter plot of citations and Altmetric scores of individual papers according to journal

# Appendix S1 – PRISMA Checklist

| **Section and Topic** | **Item #** | **Checklist item** | **Location where item is reported** |
| --- | --- | --- | --- |
| **TITLE** | | |  |
| Title | 1 | Identify the report as a systematic review. | Title page |
| **ABSTRACT** | | |  |
| Abstract | 2 | See the PRISMA 2020 for Abstracts checklist. | Abstract |
| **INTRODUCTION** | | |  |
| Rationale | 3 | Describe the rationale for the review in the context of existing knowledge. | Background |
| Objectives | 4 | Provide an explicit statement of the objective(s) or question(s) the review addresses. | Background |
| **METHODS** | | |  |
| Eligibility criteria | 5 | Specify the inclusion and exclusion criteria for the review and how studies were grouped for the syntheses. | Methods, Figure 2 Selection criteria |
| Information sources | 6 | Specify all databases, registers, websites, organisations, reference lists and other sources searched or consulted to identify studies. Specify the date when each source was last searched or consulted. | Methods |
| Search strategy | 7 | Present the full search strategies for all databases, registers and websites, including any filters and limits used. | Methods |
| Selection process | 8 | Specify the methods used to decide whether a study met the inclusion criteria of the review, including how many reviewers screened each record and each report retrieved, whether they worked independently, and if applicable, details of automation tools used in the process. | Methods, data extraction table in repository |
| Data collection process | 9 | Specify the methods used to collect data from reports, including how many reviewers collected data from each report, whether they worked independently, any processes for obtaining or confirming data from study investigators, and if applicable, details of automation tools used in the process. | Methods, data extraction table in repository |
| Data items | 10a | List and define all outcomes for which data were sought. Specify whether all results that were compatible with each outcome domain in each study were sought (e.g. for all measures, time points, analyses), and if not, the methods used to decide which results to collect. | Methods, Figure 3 Time domains |
|  | 10b | List and define all other variables for which data were sought (e.g. participant and intervention characteristics, funding sources). Describe any assumptions made about any missing or unclear information. | Methods, data extraction table in repository |
| Study risk of bias assessment | 11 | Specify the methods used to assess risk of bias in the included studies, including details of the tool(s) used, how many reviewers assessed each study and whether they worked independently, and if applicable, details of automation tools used in the process. | Methods, data extraction table in repository |
| Effect measures | 12 | Specify for each outcome the effect measure(s) (e.g. risk ratio, mean difference) used in the synthesis or presentation of results. | N/A |
| Synthesis methods | 13a | Describe the processes used to decide which studies were eligible for each synthesis (e.g. tabulating the study intervention characteristics and comparing against the planned groups for each synthesis (item #5)). | Methods |
|  | 13b | Describe any methods required to prepare the data for presentation or synthesis, such as handling of missing summary statistics, or data conversions. | Methods |
|  | 13c | Describe any methods used to tabulate or visually display results of individual studies and syntheses. | Methods |
|  | 13d | Describe any methods used to synthesize results and provide a rationale for the choice(s). If meta-analysis was performed, describe the model(s), method(s) to identify the presence and extent of statistical heterogeneity, and software package(s) used. | Methods |
|  | 13e | Describe any methods used to explore possible causes of heterogeneity among study results (e.g. subgroup analysis, meta-regression). | Methods |
|  | 13f | Describe any sensitivity analyses conducted to assess robustness of the synthesized results. | N/A |
| Reporting bias assessment | 14 | Describe any methods used to assess risk of bias due to missing results in a synthesis (arising from reporting biases). | Methods |
| Certainty assessment | 15 | Describe any methods used to assess certainty (or confidence) in the body of evidence for an outcome. | Methods |
| **RESULTS** | | |  |
| Study selection | 16a | Describe the results of the search and selection process, from the number of records identified in the search to the number of studies included in the review, ideally using a flow diagram. | Results |
|  | 16b | Cite studies that might appear to meet the inclusion criteria, but which were excluded, and explain why they were excluded. | Results |
| Study characteristics | 17 | Cite each included study and present its characteristics. | Results, Table 1, Figure 4 |
| Risk of bias in studies | 18 | Present assessments of risk of bias for each included study. | N/A |
| Results of individual studies | 19 | For all outcomes, present, for each study: (a) summary statistics for each group (where appropriate) and (b) an effect estimate and its precision (e.g. confidence/credible interval), ideally using structured tables or plots. | Results |
| Results of syntheses | 20a | For each synthesis, briefly summarise the characteristics and risk of bias among contributing studies. | Results |
|  | 20b | Present results of all statistical syntheses conducted. If meta-analysis was done, present for each the summary estimate and its precision (e.g. confidence/credible interval) and measures of statistical heterogeneity. If comparing groups, describe the direction of the effect. | Results |
|  | 20c | Present results of all investigations of possible causes of heterogeneity among study results. | N/A |
|  | 20d | Present results of all sensitivity analyses conducted to assess the robustness of the synthesized results. | N/A |
| Reporting biases | 21 | Present assessments of risk of bias due to missing results (arising from reporting biases) for each synthesis assessed. | Discussion |
| Certainty of evidence | 22 | Present assessments of certainty (or confidence) in the body of evidence for each outcome assessed. |  |
| **DISCUSSION** | | |  |
| Discussion | 23a | Provide a general interpretation of the results in the context of other evidence. | Discussion |
|  | 23b | Discuss any limitations of the evidence included in the review. | Discussion |
|  | 23c | Discuss any limitations of the review processes used. | Discussion |
|  | 23d | Discuss implications of the results for practice, policy, and future research. | Discussion |
| **OTHER INFORMATION** | | |  |
| Registration and protocol | 24a | Provide registration information for the review, including register name and registration number, or state that the review was not registered. | Open Science Framework (https://osf.io/r8vs5/) |
|  | 24b | Indicate where the review protocol can be accessed, or state that a protocol was not prepared. | Open Science Framework |
|  | 24c | Describe and explain any amendments to information provided at registration or in the protocol. | N/A |
| Support | 25 | Describe sources of financial or non-financial support for the review, and the role of the funders or sponsors in the review. | N/A |
| Competing interests | 26 | Declare any competing interests of review authors. | Competing interest |
| Availability of data, code and other materials | 27 | Report which of the following are publicly available and where they can be found: template data collection forms; data extracted from included studies; data used for all analyses; analytic code; any other materials used in the review. | Data repository |

# Appendix S2 – BIBLIO Checklist

**The BIBLIO checklist for reporting the bibliometric reviews of the biomedical literature**

| **Section/Topic** | **Item No.** | **Checklist item** | **Reported in** |
| --- | --- | --- | --- |
| **Title** |  |  |  |
| Identification | 1 | Identify the report as a bibliometric review in the title. | Introduction |
| Issues/topics | 2 | Indicate the key issues/topics under investigation and coverage of time period. | Introduction |
| **Abstract** |  |  |  |
| Structured summary | 3 | Structured summary including (as applicable): background, methods, results (key findings) and conclusions. | Abstract |
| **Introduction/ Background** |  |  |  |
| Justification/ Rationale/ Explanation | 4 | Present review of existing knowledge and epidemiological information. | Introduction |
| Objectives | 5 | Statement of the objective (s) or question (s). | Introduction |
| **Methods** |  |  |  |
| Search engines (data sources) | 6 | Describe all information sources (such as electronic databases, contact with study authors, trial registers or other grey literature sources). | Materials and Methods |
| Search strategy | 7 | Keywords and systematization criteria (date of search, language, type of document) for the search. | Appendix 1 and 2 |
| Time period | 8 | The period that the review covers and the justification. | Materials and Methods |
| Eligibility criteria | 9 | Describe all inclusion and exclusion criteria; languages; study design, type of publication and time period. | Materials and Methods, Appendix 2 |
| Data refinement (data selection procedure) | 10 | Remove the irrelevant articles; inspection to eliminate duplicate and unrelated articles (after evaluation of the title, abstract and content). | Materials and Methods |
| Quality assessment (optional) | 11 | Assessment of papers by three authors and the use of assessing checklists. | N/a |
| Data synthesis | 12 | Describe the methods used for summarizing, handling, synthesis, tabulations or schematic displays. Describe how the data were analysed. | Materials and Methods |
| **Results** |  |  |  |
| Descriptive findings (statistics) | 13 | - Provide details of the search and selection process in a flow diagram.  - Number of citations retrieved (number of publication, year of publication, type of documents, country of publication, articles with the highest impact, most impactful authors, most impactful articles, authors with the highest production, top journals, top institutions, …) | Results,  Fig1, Data repository |
| Schematic map and trend | 14 | Summarize and/or present the schematic maps and trends using an appropriate software to present citations, journals, authors, top journals, time trends, emerging literature, and any relevant indicators (as applicable) [1-5]. | Appendix,  Data repository. |
| Tabulation and summarizing the findings | 15 | General recommendation: Studies under consideration could be summarized and organized by different subtitles and different scenarios. Regardless, results need to be presented in separate tables covering each subtitle. The followings are some options that could help to summarize the findings.  *Option 1:*  - Start the presentation with a historical view [when and who first published on the topic].  - Report on review papers. The result should be listed in a separate table. Also, specify the review type (scoping review, narrative review, systematic review, and meta-analysis).  - Summarize the findings according to the study designs and main study types.  *Option 2:*  - Start the presentation with a historical view [when and who first published on the topic].  - Report on review papers. The result should be listed in a separate table. Also, indicate the review type (scoping review, narrative review, systematic review, and meta-analysis) should be specified.  - Summarize the findings according to outcome measures or populations. For example, see [6].  *Option 3:*  - Start the presentation with a historical view [when and who first published on the topic].  - Report on review papers. The result should be listed in a separate table. Also, specify the review type (scoping review, narrative review, systematic review, and meta-analysis).  - Summarize the findings according to concept [7].  *Option 4.*  - Start the presentation with a historical view [when and who first published on the topic].  - Report on review papers. The result should be listed in a separate table, and also specify the review type (scoping review, narrative review, systematic review, and meta-analysis).  - Summarize the findings according to different subtitles relevant to the main topic [8]. | Results, Figures |
| Synthesis of findings | 16 | Synthesize the findings as much as possible, find the gap, and propose a model, hypothesis, etc. (if applicable). | Results, Discussion |
| **Discussion** |  |  |  |
| Summary of evidence | 17 | Summarize the main findings. The findings should be presented in more "general" or "accessible" terms. | Discussion |
| Interpretation | 18 | Include interpretation consistent with results. Explanations for observed outcomes, similarities, and differences reported would be essential. | Discussion |
| Strengths and limitations | 19 | Discuss the strengths and limitations. | Discussion |
| Conclusion(s) | 20 | Provide a general interpretation of the results with respect to the review questions and objectives, as well as potential implications. | Discussion, Conclusion |
| 1. McDougal L, Dehingia N, Cheung WW, Dixit A, Raj A. COVID-19 burden, author affiliation and women's well-being: A bibliometric analysis of COVID-19 related publications including focus on low-and middle-income countries. eClinicalMedicine 2022; 52: 101606.  2. Henstock L, Wong R, Tsuchiya A, Spencer A. Behavioral theories that have influenced the way health state preferences are elicited and interpreted: A bibliometric mapping analysis of the ttime trade-off method with VOSviewer visualization. Front Health Serv 2022; 2: 848087.  3. Bodea F, Bungau SG, Negru AP, Radu A, Tarce AG, Tit DM, et al. Exploring new therapeutic avenues for ophthalmic disorders: Glaucoma-related molecular docking evaluation and bibliometric analysis for improved management of ocular diseases. Bioengineering 2023; 10(8): 983.  4. Sang XZ, Wang CQ, Chen W, Rong H, Hou LJ. An exhaustive analysis of post-traumatic brain injury dementia using bibliometric methodologies. Front Neurol 2023; 14: 1165059.  5. Ramli MI, Hamzaid NA, Engkasan JP, Usman J. Respiratory muscle training: a bibliometric analysis of 60 years’ multidisciplinary journey. Biomed Eng Online 2023; 22(1): 50.  6. Akosman I, Kumar N, Mortenson R, Lans A, De La Garza Ramos R, Eleswarapu A,et al. Racial differences in perioperative complications, readmissions, and mortalities after elective spine surgery in the United States: A systematic review using AI-assisted bibliometric analysis. Glob Spine J 2023: 21925682231186759.  7. Tavousi M, Mohammadi S, Sadighi J, Zarei F, Kermani RM, Rostami R, Montazeri A. Measuring health literacy: A systematic review and bibliometric analysis of instruments from 1993 to 2021. Plos One 2022; 17(7): e0271524.  8. Montazeri A. Health-related quality of life in breast cancer patients: A bibliographic review of the literature from 1974 to 2007. J Exp Clin Cancer Res 2008; 27: 32. | | | |

**Rights and permissions:** The original source of the checklist is: Montazeri A, Mohammadi S, M.Hesari P, Ghaemi M, Riazi H, Sheikhi‑Mobarakeh Z. Preliminary guideline for reporting bibliometric reviews of the biomedical literature (BIBLIO): a minimum requirements. *Systematic Reviews* 2023; 12: 239. doi.org/10.1186/s13643-023-02410-2 The article is licensed under a Creative Commons Attribution 4.0 International License (<http://creativecommons.org/licenses/by/4.0/>). A changes was made to the original checklist to add in full references to the cited sources.

# Appendix S3 – Search strings

| **Pubmed** | 2012 | ((((("American journal of respiratory and critical care medicine"[Journal]) OR ("Critical care medicine"[Journal])) OR ("Intensive care medicine"[Journal])) OR ("Critical care (London, England)"[Journal])) OR ("Annals of intensive care"[Journal])) AND (("2012/01/01"[Date - Publication] : "2012/12/31"[Date - Publication])) |
| --- | --- | --- |
|  | 2022 | ((((("American journal of respiratory and critical care medicine"[Journal]) OR ("Critical care medicine"[Journal])) OR ("Intensive care medicine"[Journal])) OR ("Critical care (London, England)"[Journal])) OR ("Annals of intensive care"[Journal])) AND (("2022/01/01"[Date - Publication] : "2022/12/31"[Date - Publication])) |
| **Scopus** | 2012 | ( SRCTITLE ( critical AND care ) OR SRCTITLE ( intensive AND care ) ) AND PUBYEAR = 2012 AND ( LIMIT-TO ( EXACTSRCTITLE , "Critical Care Medicine" ) OR LIMIT-TO ( EXACTSRCTITLE , "American Journal Of Respiratory And Critical Care Medicine" ) OR LIMIT-TO ( EXACTSRCTITLE , "Intensive Care Medicine" ) OR LIMIT-TO ( EXACTSRCTITLE , "Critical Care" ) OR LIMIT-TO ( EXACTSRCTITLE , "Annals Of Intensive Care" ) ) AND ( LIMIT-TO ( DOCTYPE , "ar" ) ) |
|  | 2022 | ( SRCTITLE ( critical AND care ) OR SRCTITLE ( intensive AND care ) ) AND PUBYEAR = 2022 AND ( LIMIT-TO ( EXACTSRCTITLE , "Critical Care Medicine" ) OR LIMIT-TO ( EXACTSRCTITLE , "American Journal Of Respiratory And Critical Care Medicine" ) OR LIMIT-TO ( EXACTSRCTITLE , "Intensive Care Medicine" ) OR LIMIT-TO ( EXACTSRCTITLE , "Critical Care" ) OR LIMIT-TO ( EXACTSRCTITLE , "Annals Of Intensive Care" ) ) AND ( LIMIT-TO ( DOCTYPE , "ar" ) ) |
| **Web of Science** | 2012 | ((((((SO=(CRITICAL CARE MEDICINE)) OR SO=(AMERICAN JOURNAL OF RESPIRATORY "AND" CRITICAL CARE MEDICINE)) OR SO=(INTENSIVE CARE MEDICINE)) OR SO=(CRITICAL CARE)) OR SO=(ANNALS OF INTENSIVE CARE)) AND PY=(2012)) AND DT=(Review OR Article OR Editorial Material OR Early Access OR Biographical-Item OR Letter OR Correction OR Bibliography OR Book Review OR Retracted Publication) |
|  | 2022 | ((((((SO=(CRITICAL CARE MEDICINE)) OR SO=(AMERICAN JOURNAL OF RESPIRATORY "AND" CRITICAL CARE MEDICINE)) OR SO=(INTENSIVE CARE MEDICINE)) OR SO=(CRITICAL CARE)) OR SO=(ANNALS OF INTENSIVE CARE)) AND PY=(2022)) AND DT=(Review OR Article OR Editorial Material OR Early Access OR Biographical-Item OR Letter OR Correction OR Bibliography OR Book Review OR Retracted Publication) |

# Appendix S4 – Inclusion and exclusion criteria

Inclusion criteria:

- original research focused on critical illness;
- published in 2022 or 2012;
- published in one of the top five ICM journals (according to SCImago at the time of search).

Exclusion criteria:

- not research, including abstracts, letters, commentaries, perspectives, opinions, case reports or editorials;
- not directly related to critical illness.

# Appendix S5 – Studies categorised according to James Lind Alliance research topics


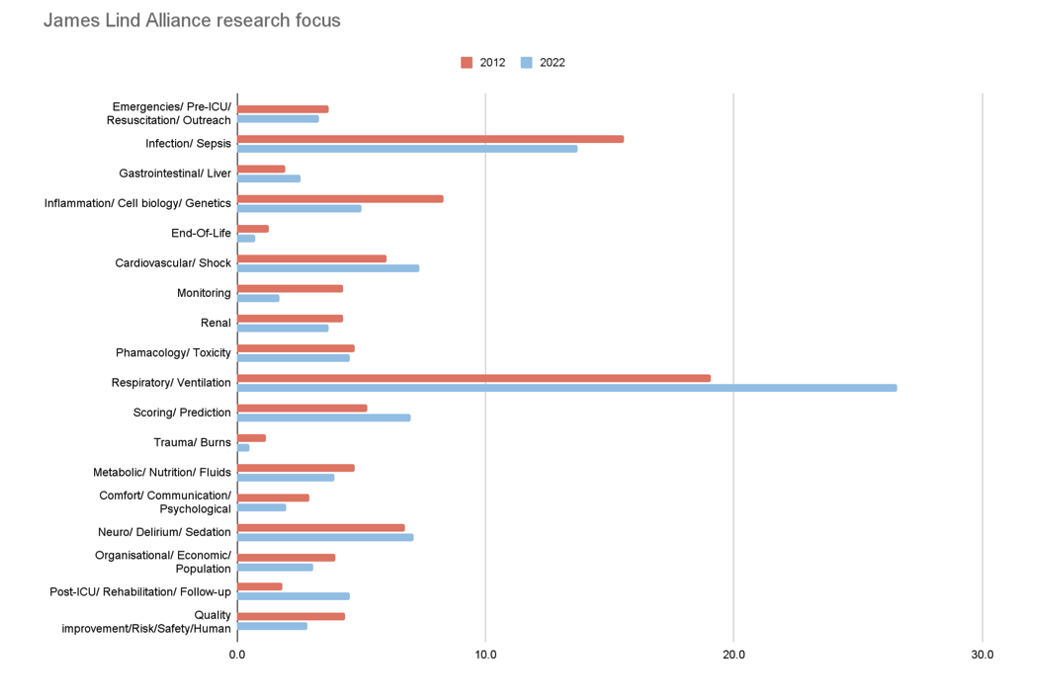


**Figure S5:** Bar chart depicting the percentage of studies (x-axis) in the top 5 ICM journals according to the SCImago ranking system that correspond with the 18 NIHR JLA research topics (y-axis). The red bars represent percentage of studies published in 2012, and the blue bars represent percentage of studies published in 2022. The topics of ‘infection/sepsis’ and ‘respiratory/ventilation’ stand out in terms of frequency.

# Appendix S6 – Citations by phases of care


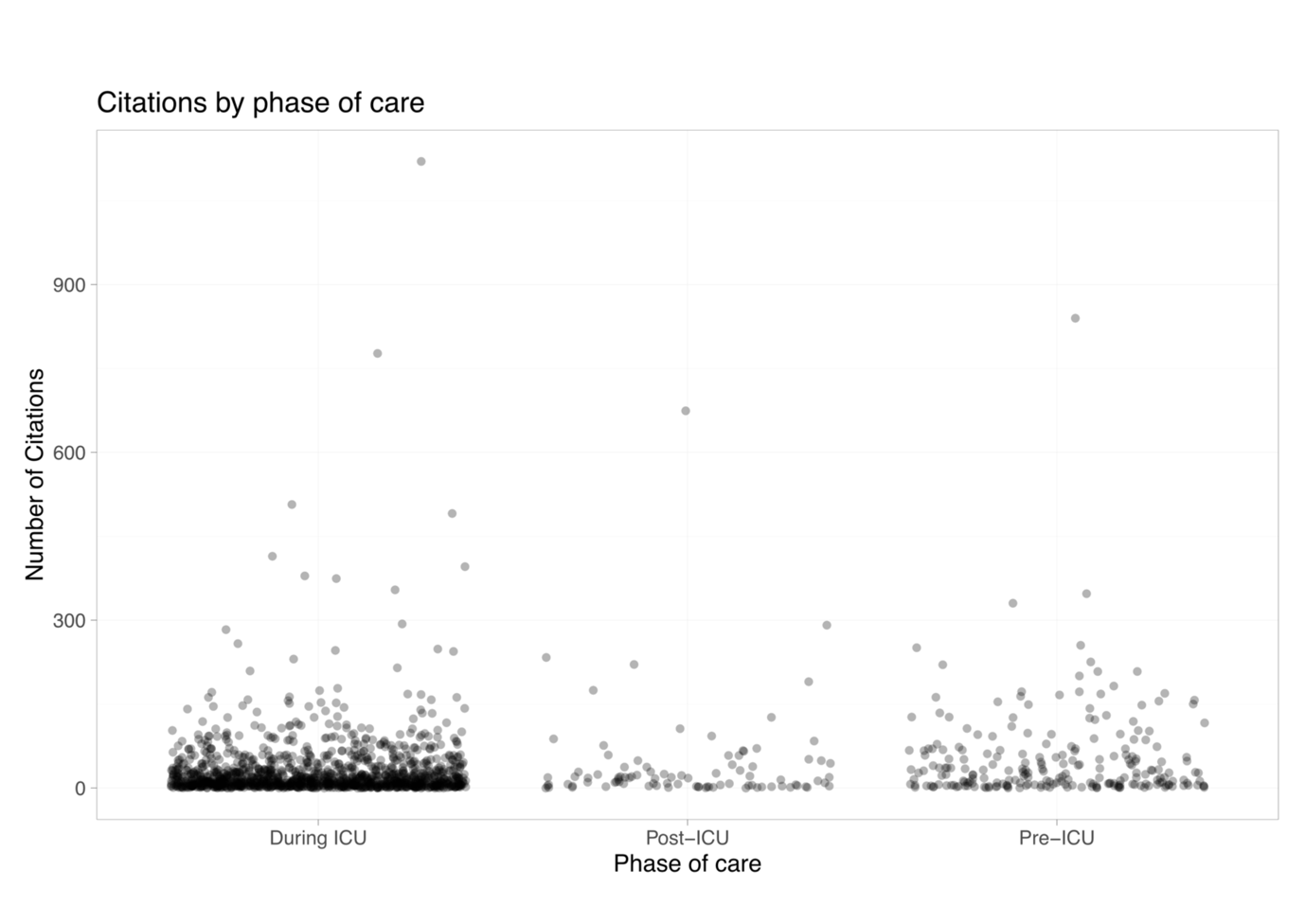


**Figure S6:** Number of citations of individual studies (dots) categorised according to the phase of care. Dense representation in the ‘in ICU’ phase of care compared with ‘post-ICU’ and ‘pre-ICU’ phases. A very small number of studies were cited extensively compared with the majority.

# Appendix S7 – Scatter plot of citations and Altmetric scores of individual papers according to journal


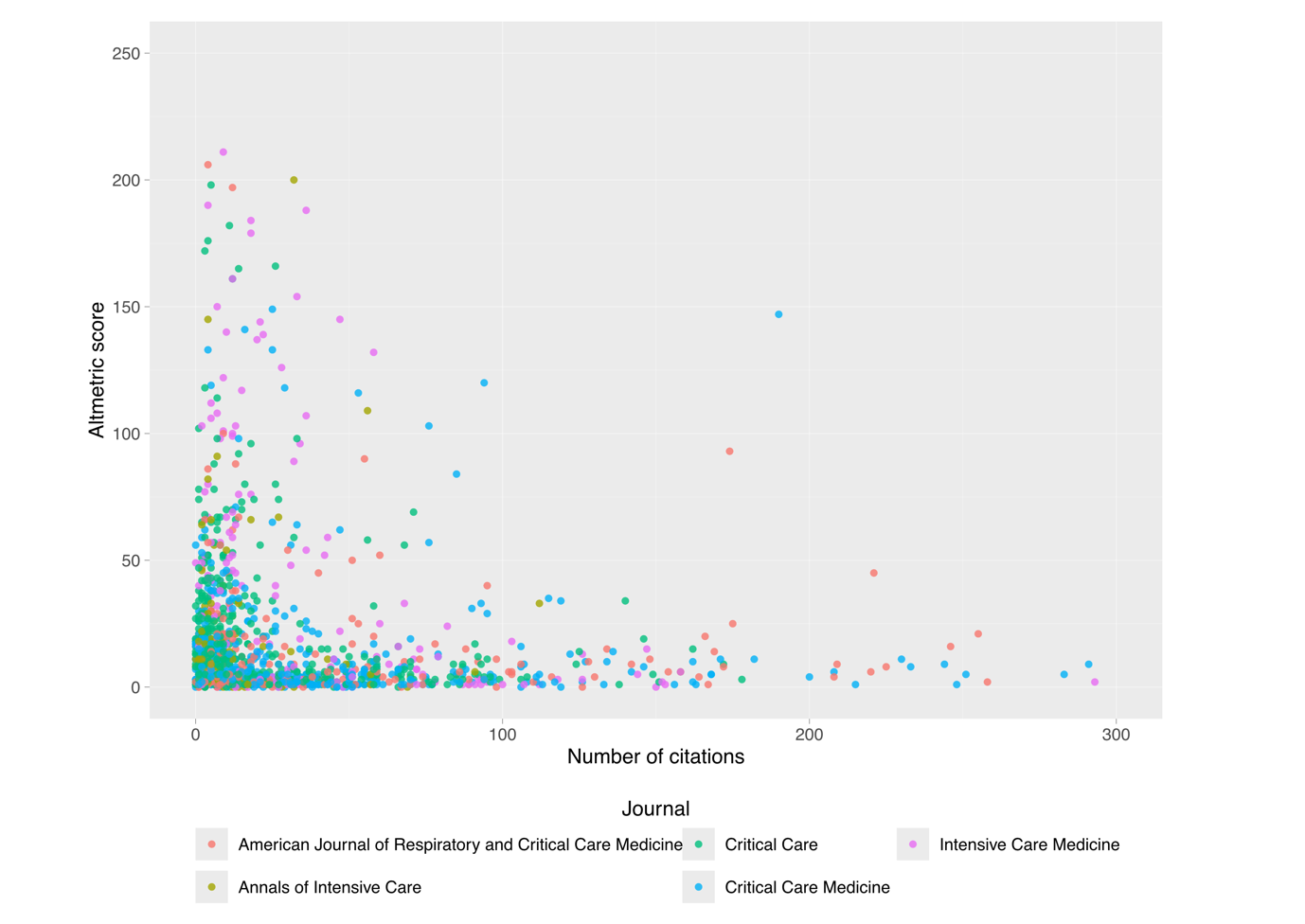


**Figure S7:** Scatter plot of studies and their corresponding number of citations (x-axis) vs Altmetric scores (y-axis). Each study is colour coded according to the journal it was published in. Most studies are balanced between number of citations and Altmetric scores. Studies with highest citation rates tend to have low Altmetric scores and vice versa.
